# Prospective Comparison of Short-Term Outcomes in Kinematic and Mechanical Alignment Total Knee Arthroplasty

**DOI:** 10.3390/clinpract15090162

**Published:** 2025-08-31

**Authors:** Ofir Vinograd, Ahmad Essa, Netanel Steinberg, Ilan Y. Mitchnik, Dana Avraham, Inon Rotem, Adi Vinograd, Yiftah Beer, Noam Shohat, Yaron Bar-Ziv

**Affiliations:** 1 Department of Orthopaedic Surgery, Shamir Medical Centre, Zerifin 7033001, Israelybarziv@gmail.com (Y.B.-Z.); 2 Faculty of Medicine, Tel Aviv University, Ramat Aviv, Tel Aviv 6997801, Israel; 3 Orthopaedic Department, Kaplan Medical Centre, Rehovot 7661041, Israel; 4 Faculty of Medicine, Bar-Ilan University, Ramat Gat 5290002, Israel; 5 The Israel Center for Disease Control, Ministry of Health, Ramat Gan 5262160, Israel

**Keywords:** total knee arthroplasty, kinematic alignment, mechanical alignment, short-term outcomes, immediate postoperative outcomes, patient reported outcome measures

## Abstract

**Background:** While mechanical alignment total knee arthroplasty (TKA) has long been the conventional surgical technique in patients with advanced osteoarthritis, kinematic alignment TKA has emerged as a promising alternative, designed to restore the knee’s native pre-arthritic anatomy. Since superiority of either technique remains inconclusive, we aimed to compare immediate and short-term postoperative outcomes of kinematic versus mechanical alignment TKA. **Methods:** This prospective cohort study was conducted at a tertiary care centre between January 2020 and August 2022, enrolling kinematic and mechanical alignment TKA patients. Outcomes were assessed during hospitalization and at 14 days postoperatively. Data collected included patient-reported outcome measures (PROMs), functional performance evaluations, pain scores, discharge disposition and hospital length of stay. Both univariate and multivariate regression analyses were conducted, adjusting for potential confounders. **Results:** The study included 103 patients, with 77 who underwent kinematic alignment and 26 mechanical alignment TKA. Patients in the kinematic alignment group demonstrated statistically significant better postoperative outcomes compared to those in the mechanical alignment group. Kinematic alignment TKA patients demonstrated superior functional performance on the Timed Up and Go test immediately postoperatively and were more frequently discharged home rather than to a rehabilitation facility. Hospital stay length and short-term PROMs also favoured the Kinematic alignment TKA group, showing statistically significant higher scores in the Oxford Knee Score, short form-12 Mental Component Summary, and the Knee Injury and Osteoarthritis Outcome Score Symptoms subscale. **Conclusions:** Kinematic alignment TKA offers superior immediate and short-term outcomes compared to mechanical alignment TKA, with benefits in functional recovery, hospitalization duration, and discharge disposition. This evidence supports kinematic alignment TKA as a viable alternative, aiding in patient and surgeon decision-making.

## 1. Introduction

Total knee arthroplasty (TKA) is a widely performed surgical procedure aimed at relieving pain and improving quality of life in patients with advanced osteoarthritis that has failed conservative treatments [1,2]. The growing demand for TKA is evident, with projections suggesting that by 2030, approximately 3.5 million TKA procedures will be performed annually in the U.S., marking a 680% increase since 2005 [3].

Given the increasing demand for TKA, the development and optimization of surgical techniques have become critical. Mechanical alignment (MA) TKA, the conventional approach, seeks to create a mechanically aligned limb through precise bone cuts and soft tissue balancing [1]. This method is well-established as a safe and effective treatment for end-stage osteoarthritis [4]. However, despite considered highly effective, 15–20% of patients report dissatisfaction with their postoperative outcomes, prompting the exploration of alternative approaches [5]. Kinematic alignment (KA) TKA has emerged as a dominant alternative. It aims to restore the knee pre-arthritic anatomy using bone cuts with minimal soft tissue manipulation [6].

Several randomized controlled trials and meta-analyses reported the high safety and effectivity of MA and KA TKA, though superiority of either technique remains inconclusive [7,8,9,10,11,12,13,14,15]. Most studies, however, have concentrated on medium- and long-term outcomes, leaving a gap in knowledge regarding short-term recovery and postoperative rehabilitation.

The current study aims to compare objective and patient-reported immediate and short-term postoperative outcomes between patients undergoing KA and MA TKA.

## 2. Materials and Methods

### 2.1. Study Design and Patient Selection

**Figure 1:**
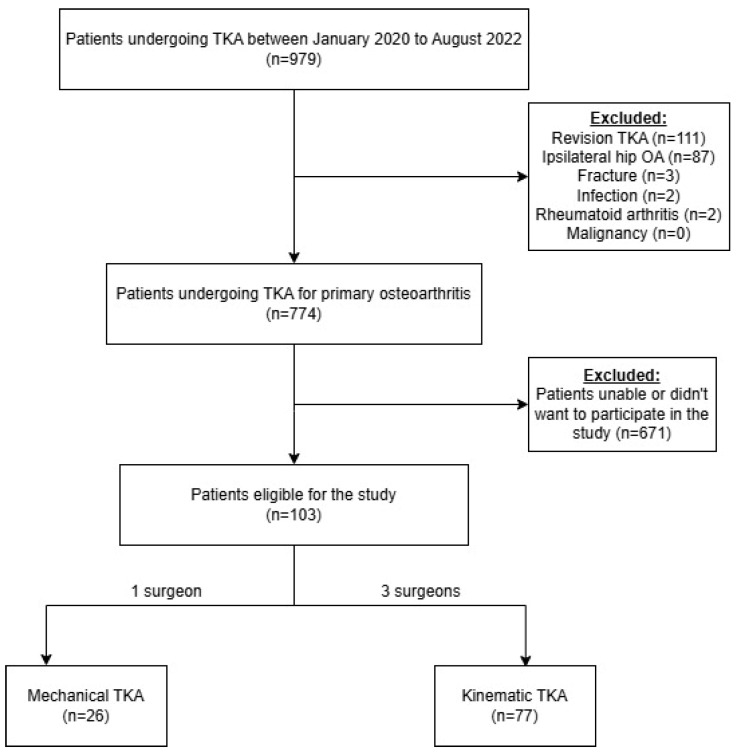
Designation of individuals to study groups. Abbreviations: TKA = total knee arthroplasty; OA = osteoarthritis. A flowchart illustrating the patient selection process for each group.

This prospective cohort study, conducted at a tertiary care institution, included all patients who underwent TKA using either MA or KA techniques between January 2020 and August 2022. Inclusion criteria consisted of patients classified as Kellgren–Lawrence grade 3 or 4 [16], who had failed non-operative management, including structured physiotherapy and intra-articular corticosteroid injections. Exclusion criteria were revision surgeries, concomitant ipsilateral symptomatic hip osteoarthritis, TKA due to fracture, infection, rheumatoid arthritis, and malignancy (Figure 1). Eligible patients were asked to provide informed consent to participate in the study. Patients were scheduled for surgery either to a single surgeon performing MA TKA or to one of three surgeons performing KA TKA (Figure 1). All surgeons were high-volume practitioners (>100 procedures per year), each with over 10 years of experience. Patients were followed during hospitalization and at 14 days postoperatively (POD 14) for several outcomes: (1) patient-reported outcome measures (PROMs); (2) functional performance; (3) pain relief and analgesic use; (4) length of hospitalization; and (5) discharge disposition. The study protocol received approval from the local institutional review board.

#### Data Collection

Baseline patient characteristics, hospitalization details, and surgical data were extracted from computerized patient records. These included age, sex, body mass index (BMI), smoking status, baseline health status, American Society of Anaesthesiologists (ASA) classification, anaesthesia type (general or spinal), peripheral nerve block (given or not), operated side, hospitalization length, discharge disposition and return to the emergency room (ER) due to pain within two weeks. Prospective data collection included Timed Up and Go (TUG) and Visual Analogue Scale (VAS) on the first postoperative day (POD 1). Discharge timing and disposition were determined based on standardized medical, functional, and social criteria, and were applied uniformly to all patients, irrespective of the arthroplasty technique used or the operating surgeon. Functional performance tests were assessed and record at scheduled postoperative clinic visits using the TUG test and the stairs test. In addition, PROMs questionnaires were recorded preoperative and at POD 14. The functional assessments and PROMs questionnaires were conducted by a physician blinded to surgery type.

### 2.2. Variable Definitions

Baseline health status was measured by the Charlson Comorbidity Index (CCI). PROMs included the VAS, Knee Injury and Osteoarthritis Outcome Score (KOOS), Forgotten Joint Score (FJS), Oxford Knee Score (OKS), and Short Form-12 (SF-12) questionnaires [17,18,19]. The TUG test measured the time required for a patient to rise from a chair, walk 3 m, turn, return, and sit back down, while the stairs test measured the time taken to ascend and descend a flight of nine stairs [20].

### 2.3. Surgical and Rehabilitation Techniques

All surgeries were performed using a midline incision with a medial parapatellar approach [21]. KA TKA was performed using the “calipered kinematic” technique, while MA TKA was performed using the standard gap balancing technique [22,23]. All patients underwent preoperative standing anteroposterior and lateral knee radiographs and EOS imaging. No formal templating was performed, and alignment was determined intraoperatively based on established surgical protocols. Femoral and tibial implants were cemented in both groups. Implant types included either cruciate-retaining (CR) or cruciate-sacrificing (CS) designs, selected based on the integrity of the posterior cruciate ligament, which was assessed preoperatively and/or intraoperatively by the operating surgeon. The postoperative analgesic regimen was standardized across groups and included Acetaminophen (1 g every 8 h), Dipyrone (1 g every 8 h), Etodolac (400 mg twice daily, limited for patients without renal impairment), and Gabapentin (300 mg twice daily). Opioids were administered upon request if patients reported severe pain (VAS > 8) despite the baseline analgesic regimen [24], with options including Oxycodone (5 mg or 10 mg) and Tramadol (100 mg) [24]. Opioid use was converted to Morphine Equivalent Dose for standardization [25]. Physiotherapy commenced on POD 1 in both groups, with patients encouraged to begin full weight-bearing and a range of motion exercises as tolerated [26].

### 2.4. Statistical Analysis

Descriptive statistics are reported as mean and standard deviation for continuous normally distributed variables and as percentages for categorical variables. Continuous variables were assessed using the Mann–Whitney U test, while categorical variables were assessed with the Chi-square test. Multivariate regression models were employed using linear regression for continuous outcomes and logistic regression for binary outcomes. Mean differences (MD) and odds ratios (OR) with 95% confidence intervals (CI) were reported for linear and logistic regression, respectively. The models were adjusted for patient and surgery-related potential confounders, in addition to variables that were statistically significant in the univariate analyses. Specifically, models accounted for age, sex, BMI, anaesthesia type, peripheral nerve block, baseline health status, and performance on the stairs test. A statistically significant difference was assumed when *p*-value ≤ 0.05. All statistical analyses were conducted using IBM SPSS Statistics for Windows, Version 23.0 (IBM Corp., Armonk, NY, USA).

## 3. Results

The study cohort included 103 patients who underwent TKA, with 77 patients in the KA TKA group and 26 in the MA TKA group, reflecting the proportion of surgeons performing each technique. Baseline characteristics of study population by TKA type are presented in Table 1. The mean age was 69.3 ± 8.4 years, with a female predominance (63.1%), both comparable between study groups. The mean BMI was also similar between groups, averaging 31.8 ± 6.5 kg/m^2^. Most implants were of a CR design, with no statistically significant difference between groups (*p* = 0.665). In opposed to preoperative measures that were comparable between study groups (pain VAS, TUG, OKS, SF-12, and KOOS), a statistically significant difference was noted in the preoperative stairs climb test favouring the KA TKA group (29.9 ± 21.4 s vs. 46.0 ± 23.7 s, *p* = 0.003). No statistically significant differences were observed among the three surgeons performing KA TKA.

### 3.1. Hospitalization and POD 1 Outcomes

**Figure 2:**
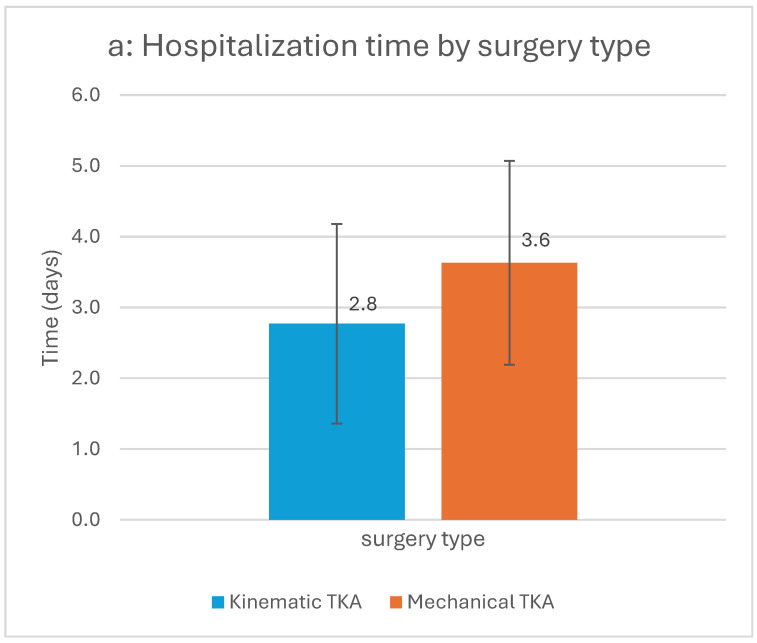

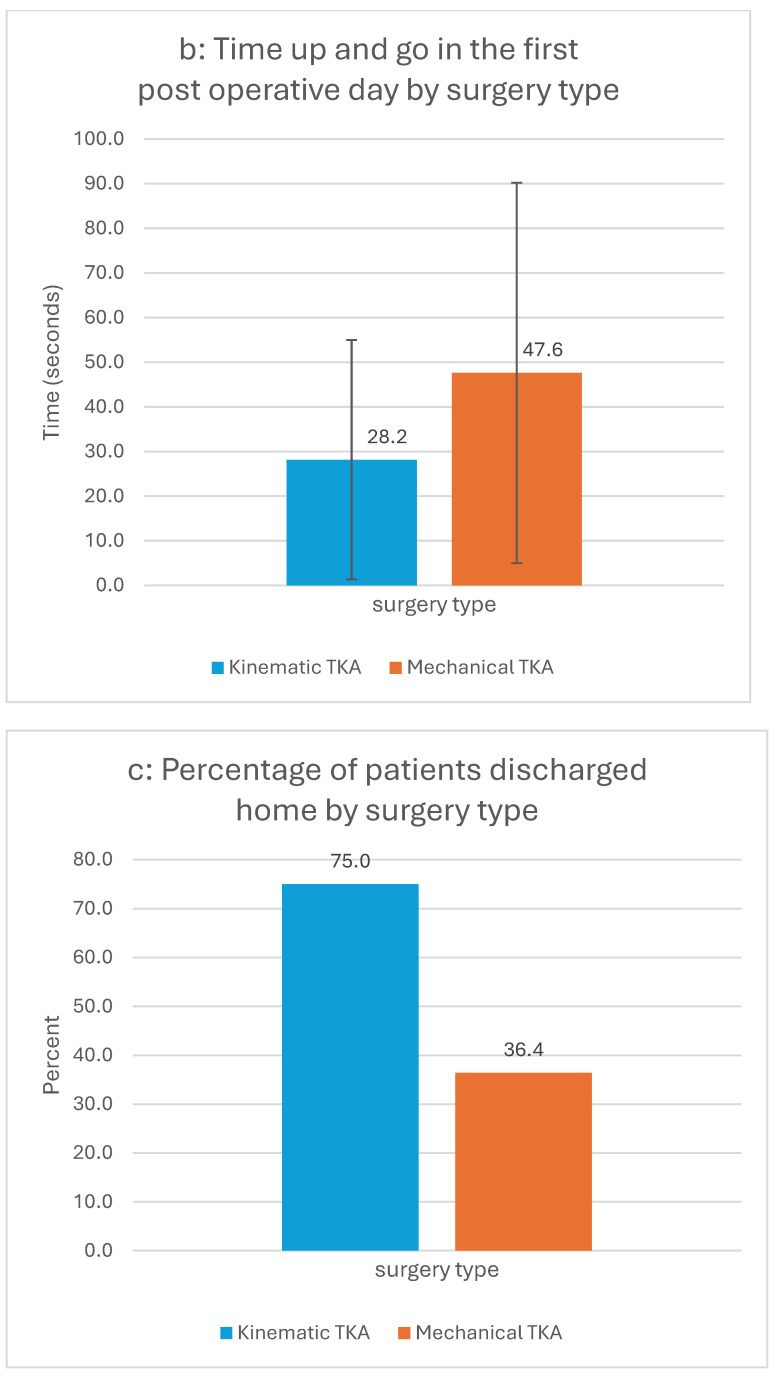
(**a**–**c**) Statistically significant differences between study groups in hospitalization outcome parameters. Abbreviations: TKA = total knee arthroplasty; TUG = time up and go.

Patients in the KA TKA group had a shorter hospitalization duration (2.77 vs. 3.63 days, *p* = 0.013) and were more frequently discharged home rather than to a rehabilitation facility compared to the MA TKA group (75% vs. 36.4%, *p* = 0.001) (Figure 2). There was no statistically significant difference in opioid use during hospitalization, either in total consumption or day-to-day comparisons, between KA and MA groups. Similarly, there was no statistically significant difference in POD 1 pain scores on the VAS between the groups (Table 2).

Patients in the KA TKA group had shorter hospitalization time (a), showed faster times in TUG test (b) and were more likely to be discharged home compared to MA TKA patients (c). The two-sided bars in Figure 2a,b represent the 95% confidence intervals. *p*-value < 0.05 for all three univariable analyses.

**Figure 3:**
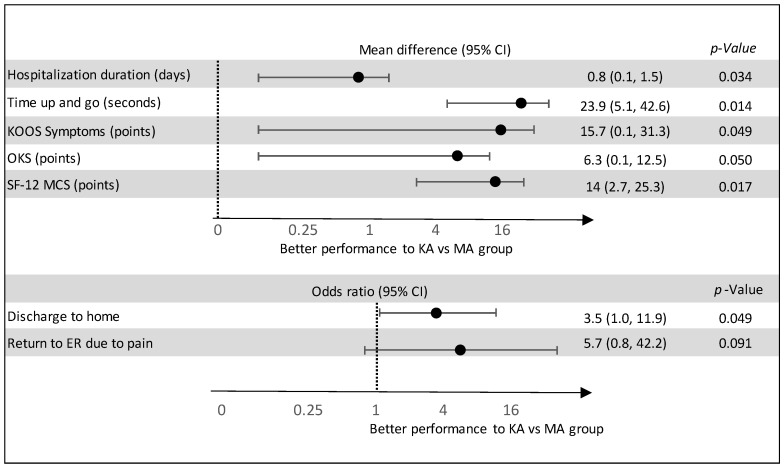
Multivariate regression analysis for hospitalization and post discharge variables. Abbreviations: POD 14 = post operative day 14; MA = mechanical alignment; CI = confidence interval; KOOS = knee injury and osteoarthritis outcome score; OKS = Oxford knee score; SF-12 MCS = Short form-12 mental component score; KA = kinematic alignment; ER = emergency room.

In terms of functional performance, the TUG test on POD 1 showed statistically significant favorable results in the KA group compared to the MA group (28.2 ± 26.8 vs. 47.6 ± 42.6 s, *p* = 0.023) (Figure 2). Findings persisted in multivariable analysis, where the KA TKA group demonstrated statistically significant better outcomes for POD 1 TUG (MD = 23.9, 95% CI 5.1 to 42.6) and hospitalization duration (MD 0.76, 95% CI −1.47 to −0.06, indicating shorter hospitalization for the KA group. Furthermore, patients in the KA TKA group were approximately 3.5 times more likely to be discharged home, rather to a rehabilitation facility, compared to the MA TKA group (OR = 3.5, 95% CI 1.01–11.96) (Figure 3). 

The forest plot demonstrates superior outcomes for patients in the KA TKA group relative to those in the MA group. The x-axis is presented on a logarithmic scale. The upper section of the figure depicts the mean difference, with values greater than zero indicating better performance for the KA TKA group. The lower section displays odds ratios, where values exceeding one signify better outcomes for the KA TKA group.

### 3.2. POD 14 Outcomes

At POD 14, univariate analysis of PROMs showed statistically significant better outcomes in the KA TKA group compared to the MA TKA group, particularly in OKS (14.2 vs. 9.4, *p* = 0.034), SF-MCS (40.1 vs. 22.1, *p* < 0.001), and KOOS-Symptoms (42.6 vs. 28.7, *p* = 0.033) (Table 2). The return rate to the ER due to pain, the KOOS-Function and Daily Living, and VAS were found to be not statistically significant between groups (Table 2).

Multivariable models confirmed statistically significant superior outcomes for KA TKA at POD 14, particularly in KOOS-Symptoms (MD 15.7, 95% CI 0.08 to 31.3), OKS (MD 6.3, 95% CI 0.01 to 12.5), and SF-12 MCS (MD 14, 95% CI 2.7 to 25.3). The rate of return to the ER due to pain remained non-statistically significant following adjustment to baseline characteristics (Figure 3). Notably, none of the patients required re-hospitalization or revision surgery within the two-week postoperative period, nor experienced any significant surgery-related adverse events.

### 3.3. Sensitivity Analysis of Loss to Follow-Up

The loss to follow-up rate at POD 14 was approximately 15% in the MA TKA group and 30% in the KA TKA group. Sensitivity analysis showed that preoperative patient characteristics were generally comparable between those attended and those who did not attend the POD 14 evaluation (Supplementary Tables S1 and S2). However, within the KA group, patients who did not attend the POD 14 evaluation demonstrated faster preoperative stair test completion compared to those who attended (*p* = 0.013) (Supplementary Table S1).

## 4. Discussion

In this prospective cohort study comparing MA and KA TKA, patients who underwent KA TKA experienced statistically significant improvements in short-term outcomes. These included better performance in the TUG test immediately postoperatively, shorter hospital stays, and a higher likelihood of being discharged home instead of to a rehabilitation facility. Importantly, there were no statistically significant differences between groups regarding pain or opioid use during hospitalization. The superior outcomes in the KA TKA group persisted throughout the short-term follow-up, as demonstrated by several PROMs. These results remained consistent following adjustment to baseline characteristics that were suspected as potential confounders.

Our findings align with previous studies reporting improved PROMs in KA TKA patients [14,15]. However, while prior research has focused on longer-term outcomes, our study highlights statistically significant differences in short-term postoperative outcomes, such as improvements in KOOS-Symptoms, OKS, and SF12-MCS scores. These differences exceeded the minimal clinically important difference, underscoring their clinical significance [27].

The enhanced short-term outcomes in the KA TKA group may be attributed to reduced soft tissue manipulation and the preservation of pre-arthritic joint alignment, which could lead to better proprioception, stability, and more natural knee kinematics [28,29]. A recent study by Pintore et al. [30] demonstrated that KA TKA preserves native limb alignment measurements, with no significant pre- to postoperative changes, and maintains a joint line parallel to the ground on standing radiographs. These factors might contribute to a faster functional recovery observed in the KA group compared to MA TKA group.

From a healthcare economics perspective, the shorter hospital stays and higher rates of discharge home in the KA group could reduce the overall costs associated with TKA procedures and support the feasibility of KA TKA for outpatient surgery [31]. Additionally, although a higher rate of ER readmissions due to pain was noted in the MA TKA group, the absolute number of readmissions was low, suggesting that our study might have been underpowered to detect a statistically significant difference. Further research is needed to confirm these findings and evaluate their broader impact on healthcare systems and costs.

### 4.1. Limitations

This study has several limitations. First, the loss to follow-up at POD 14 may have introduced a selection bias. However, sensitivity analysis demonstrated that the only statistically significant difference between those who completed follow-up to those who did not, was the preoperative stairs test among patients in the KA TKA group, minimizing the likelihood of significant bias. Moreover, national patient records confirmed that none of the patients lost to follow-up experienced serious adverse events. Second, the non-randomized, single-centre design may limit generalizability, although the inclusion of multiple surgeons for KA TKA enhances the external validity of our findings. Third, while all surgeons were high-volume practitioners with substantial experience, variations in surgical technique or preferences may have introduced surgeon bias. Nevertheless, surgical parameters were not statistically different between surgeons or study groups, mitigating the potential impact of this bias. Fourth, the relatively small sample size of the MA TKA group may have limited the statistical power to detect true differences between groups. Nevertheless, the statistically significant differences identified in the current cohort underscore the superiority of KA TKA over MA TKA in the immediate and short-term postoperative period. Finally, as with any observational study, there remains the potential for unmeasured confounding variables that could have influenced the outcomes, despite our efforts to control for known baseline characteristics.

### 4.2. Strengths

This study has several notable strengths. First, preoperative characteristics were largely comparable between the study groups. Additionally, the use of multivariable analyses, to adjust for potential confounders, further supports the robustness of the findings favouring KA TKA. Second, the collection of data by a physician blinded to surgery type enhances the internal validity of the results. Third, the sensitivity analysis addressing loss to follow-up indicates that missing data was unlikely to have introduced bias into the study findings. Fourth, the study’s outcomes—evaluating both objective performance tests and PROMs—offers a comprehensive assessment of recovery. The PROMs encompass both physical and mental health domains, aligning with a patient-centred care approach.

## 5. Conclusions

This study demonstrates that KA TKA yields statistically significant superior short-term outcomes compared to MA TKA, as evidenced by both objective measures and PROMs. The short-term advantages of KA TKA, including expedited functional recovery and higher discharge rates to home, provide compelling evidence to support its broader adoption in clinical practice.

## Figures and Tables

**Table 1:** Baseline clinical and demographic characteristics of patients by study groups.

Variable	Kinematic TKA (N = 77)	Mechanical TKA (N = 26)	*p*-Value
Age, years (mean ± SD)	68.0 ± 8.6	71.0 ± 7.6	0.312
Sex, female (%)	62.3	65.4	0.781
BMI, kg/m^2^ (mean ± SD)	31.8 ± 6.8	31.5 ± 5.4	0.835
Side operated, Left (%)	45.0	63.6	0.135
Implant design, Cruciate Retaining (%)	84.4	80.8	0.665
Smoker (%)	11.7	11.5	0.984
ASA (%)			0.944
1	3.0	4.5	
2	48.5	45.5	
3	48.5	50.0	
CCI (%)			0.652
No or mild	32.4	22.7	
Moderate	55.9	68.2	
Severe	11.8	9.1	
Preoperative VAS (mean ± SD)	8.3 ± 1.4	8.5 ± 1.2	0.673
Preoperative TUG, seconds (mean ± SD)	19.5 ± 9.4	23.7 ± 10.6	0.064
Preoperative Stairs climb, seconds (mean ± SD)	29.9 ± 21.4	46.0 ± 23.7	0.003
Preoperative OKS (mean ± SD)	16.4 ± 7.7	14.9 ± 7.2	0.436
Preoperative SF 12 (mean ± SD)			
PCS	27.9 ± 7.8	27.6 ± 6.4	0.973
MCS	47.0 ± 12.6	43.0 ± 9.0	0.129
Preoperative KOOS (mean ± SD)			
Overall	31.3 ± 12.9	26.1 ± 11.2	0.068
Symptoms	47.0 ± 12.6	43.0 ± 9.1	0.077
Pain	36.8 ± 18.8	31.5 ± 15.4	0.194
Function	38.2 ± 18.2	31.2 ± 14.4	0.103
Quality of Life	17.8 ± 14.0	15.3 ± 13.3	0.553
Sports	15.7 ± 19.5	11.7 ± 19.4	0.229
Preoperative FJS (mean ± SD)	9.5 ± 7.9	9.2 ± 5.6	0.088

Abbreviations: TKA = total knee arthroplasty; SD = standard deviation; BMI = body mass index; ASA = American society of anaesthesiologists; CCI = Charlson comorbidity index; VAS = visual analogue scale; OKS = Oxford knee score; SF 12 = Short form 12; PCS = physical component score; MCS = mental component score; KOOS = knee injury and osteoarthritis outcome score; FJS = forgotten joint score.

**Table 2:** Hospitalization and post operative day 14 outcomes of patients by study group.

Variable	Kinematic TKA (N = 54)	Mechanical TKA (N = 23)	*p*-Value
**Hospitalization**			
Hospitalization duration, days (mean ± SD)	2.8 ± 1.4	3.6 ± 1.4	0.013
Discharge to home (%)	75.0	36.4	0.001
Total opioid use (Morphine Equivalent Dose) (mean ± SD)	58.9 ± 47.5	78.7 ± 67.3	0.224
**POD 1**			
TUG, seconds (mean ± SD)	28.2 ± 26.8	47.6 ± 42.6	0.023
VAS (mean ± SD)	5.9 ± 4.1	5.8 ± 4.1	0.970
**POD 14**			
TUG, seconds (mean ± SD)	22.7 ± 20.2	20.7 ± 19.8	0.774
Stairs test, seconds (mean ± SD)	55.3 ± 60.1	52.6 ± 77.7	0.680
VAS (mean ± SD)	5.9 ± 3.2	4.7 ± 3.8	0.192
OKS (mean ± SD)	14.2 ± 9.9	9.4 ± 10.5	0.034
SF 12 MCS (mean ± SD)	40.1 ± 19.2	22.1 ± 18.0	<0.001
KOOS Symptoms (mean ± SD)	42.6 ± 25.3	28.7 ± 26.6	0.033
KOOS Function and Daily Living (mean ± SD)	35.4 ± 24.6	25.5 ± 25.1	0.093
Return to ER due to pain (%)	4.2	18.2	0.052

Abbreviations: POD 14 = post operative day 14; TKA = total knee arthroplasty; SD = standard deviation; TUG = time up and go test; VAS = visual analogue scale; OKS = Oxford knee score; SF 12 = Short form 12; MCS = mental component score; KOOS = knee injury and osteoarthritis outcome score; ER = emergency room. Boldface is used to denote timeframes for clarity.

## Data Availability

The data presented in this study is available on request from the corresponding author due to restrictions imposed by the Institutional Review Board (IRB); access to the data requires prior authorization from the IRB.

## Supplementary Materials

The following supporting information can be downloaded at: https://www.mdpi.com/article/10.3390/clinpract15090162/s1, Table S1: Baseline clinical and demographic characteristics of patients in the kinematic alignment group by lost to follow up status on post operative day 14; Table S2: Baseline clinical and demographic characteristics of patients in the mechanical alignment group by lost to follow up status on post operative day 14.
