# Supplementary material for: Prospective Comparison of Short-Term Outcomes in Kinematic and Mechanical Alignment Total Knee Arthroplasty
Source: Clin Pract. 2025 Aug 31;15(9):162. doi: 10.3390/clinpract15090162 (PMC12468619; doi:10.3390/clinpract15090162)
Supplement: Supplementary file 1 [file clinpract-15-00162-s001.zip › clinpract-3751602-supplementary.pdf]

Table S1. Baseline clinical and demographic characteristics of patients in the kinematic alignment group by lost to follow up status on post operative day 14**.**

Variable	Attended POD 14 evaluation (n=54)	Lost to follow up at POD 14 (n=23)	P-value
Age, years (mean ± SD)	69.0 ± 8.0	68.0 ± 9.0	0.602
Sex, female (%)	64.8	60.9	0.616
BMI, kg/m^2 (mean ± SD)	32.0 ± 6.0	32.0 ± 8.0	0.499
Side operated, Left (%)	44.4	47.8	0765
Smoker (%)	5.6	17.4	0.058
ASA (%)			0.166
1	3.7	0.0	
2	55.6	17.4	
3	40.7	82.6	
CCI (%)			0.251
No or mild	25.9	17.4	
Moderate	61.1	78.3	
Severe	13.0	4.3	
Preoperative VAS (mean ± SD)	8.0 ± 2.0	8.0 ± 1.0	0.987
Preoperative TUG, seconds (mean ± SD)	20.6 ± 9.0	17.8 ± 9.9	0.111
Preoperative Stairs climb, seconds (mean ± SD)	35.4 ± 22.9	19.6 ± 13.6	0.013
Preoperative OKS (mean ± SD)	17.1 ± 7.1	15.7 ± 8.2	0.391
Preoperative SF12 (mean ± SD)			
PCS	29.6 ± 7.8	26.0 ± 7.6	0.637
MCS	45.8 ± 12.1	48.3 ± 13.1	0.353
Preoperative KOOS (mean ± SD)			
Overall	34.1 ± 12.4	28.4 ± 13.1	0.059
Symptoms	47.1 ± 14.5	46.8 ± 22.6	0.878
Pain	40.7 ± 16.2	32.7 ± 20.8	0.141
Function	41.3 ± 16.7	34.7 ± 19.4	0.074
Quality of Life	20.7 ± 14.4	14.7 ± 13.1	0.078
Sports	21.5 ± 18.5	16.8 ± 11.5	0.115
Preoperative FJS (mean ± SD)	9.0± 6.2	9.7 ± 9.5	0.185

Abbreviations: TKA = total knee arthroplasty; SD = standard deviation; BMI = body mass index; ASA = American society of anaesthesiologists; CCI = Charlson comorbidity index; VAS = visual analogue scale; OKS – Oxford knee score; SF 12 = Short form 12; PCS = physical component score; MCS = mental component score; KOOS = knee injury and osteoarthritis outcome score; FJS = forgotten joint score.

Table S2. Baseline clinical and demographic characteristics of patients in the mechanical alignment group by lost to follow up status on post operative day 14.

Variable	Attended POD 14 evaluation (n=22)	Lost to follow up at POD 14 (n=4)	P-value
Age, years (mean ± SD)	69.0 ± 7.0	73.0 ± 8	0.180
Sex, female (%)	72.7	50.0	0.320
BMI, kg/m^2 (mean ± SD)	31.0 ± 6.0	32.0 ± 5.0	0.609
Side operated, Left (%)	59.1	75.0	0.604
Smoker (%)	18.2	25.0	0.115
ASA (%)			0.746
1	9.1	0.0	
2	45.4	50.0	
3	45.4	50.0	
CCI (%)			0.197
No or mild	31.8	25.0	
Moderate	54.5	50.0	
Severe	13.7	25.0	
Preoperative VAS (mean ± SD)	9.0 ± 1.0	8.0 ± 1.0	0.141
Preoperative TUG, seconds (mean ± SD)	22.6 ± 11.0	25.5 ± 10.1	0.558
Preoperative Stairs climb, seconds (mean ± SD)	43.6 ± 24.3	50.0 ± 22.5	0.407
Preoperative OKS (mean ± SD)	16.6 ± 6.8	12.6 ± 7.4	0.148
Preoperative SF12 (mean ± SD)			
PCS	29.6 ± 6.3	25.0 ± 6	0.054
MCS	43.2 ± 8.5	42.0 ± 10.0	0.683
Preoperative KOOS (mean ± SD)			
Overall	27.2 ± 11.1	25.0 ± 11.7	0.540
Symptoms	39.6 ± 19.3	36.9 ± 21.0	0.878
Pain	31.5 ± 15.7	31.5 ± 15.8	0.878
Function	32.2 ± 16.0	29.7 ± 12.6	0.610
Quality of Life	20.3 ± 11.9	12.5 ± 8	0.200
Sports	20.6 ± 13.7	18.0 ± 9.0	0.646
Preoperative FJS (mean ± SD)	9.7 ± 7.3	8.0 ± 3.0	0.134

Abbreviations: TKA = total knee arthroplasty; SD = standard deviation; BMI = body mass index; ASA = American society of anaesthesiologists; CCI = Charlson comorbidity index; VAS = visual analogue scale; OKS – Oxford knee score; SF 12 = Short form 12; PCS = physical component score; MCS = mental component score; KOOS = knee injury and osteoarthritis outcome score; FJS = forgotten joint score.
